# Case report: Refractory Evans syndrome in two patients with spondyloenchondrodysplasia with immune dysregulation treated successfully with JAK1/JAK2 inhibition

**DOI:** 10.3389/fimmu.2023.1328005

**Published:** 2024-01-29

**Authors:** Yael Gernez, Mansi Narula, Alma-Martina Cepika, Juanita Valdes Camacho, Elisabeth G. Hoyte, Kirsten Mouradian, Bertil Glader, Deepika Singh, Bindu Sathi, Latha Rao, Ana L. Tolin, Kenneth I. Weinberg, David B. Lewis, Rosa Bacchetta, Katja G. Weinacht

**Affiliations:** ^1^ Division of Allergy, Immunology and Rheumatology, Department of Pediatrics, Stanford School of Medicine, Stanford, CA, United States; ^2^ Division of Hematology, Oncology, Stem Cell Transplantation and Regenerative Medicine, Department of Pediatrics, Stanford School of Medicine, Stanford, CA, United States; ^3^ Division of Allergy and Immunology, Department of Pediatrics, Louisiana State University (LSU) Health, Shreveport, LA, United States; ^4^ Division of Rheumatology, Department of Pediatrics, Valley Children Hospital, Madera, CA, United States; ^5^ Division of Hematology, Department of Pediatrics, Valley Children Hospital, Madera, CA, United States; ^6^ Division of Immunology, Department of Pediatrics, Hospital Pediatrico Dr. Humberto Notti, Mendoza, Argentina

**Keywords:** spondyloenchondrodysplasia, ACP5, tartrate-resistant acid phosphatase, autoimmunity, interferonopathy, JAK inhibitor, myeloma

## Abstract

Biallelic mutations in the *ACP5* gene cause spondyloenchondrodysplasia with immune dysregulation (SPENCDI). SPENCDI is characterized by the phenotypic triad of skeletal dysplasia, innate and adaptive immune dysfunction, and variable neurologic findings ranging from asymptomatic brain calcifications to severe developmental delay with spasticity. Immune dysregulation in SPENCDI is often refractory to standard immunosuppressive treatments. Here, we present the cases of two patients with SPENCDI and recalcitrant autoimmune cytopenias who demonstrated a favorable clinical response to targeted JAK inhibition over a period of more than 3 years. One of the patients exhibited steadily rising IgG levels and a bone marrow biopsy revealed smoldering multiple myeloma. A review of the literature uncovered that approximately half of the SPENCDI patients reported to date exhibited increased IgG levels. Screening for multiple myeloma in SPENCDI patients with rising IgG levels should therefore be considered.

## Introduction

**Figure 1:**
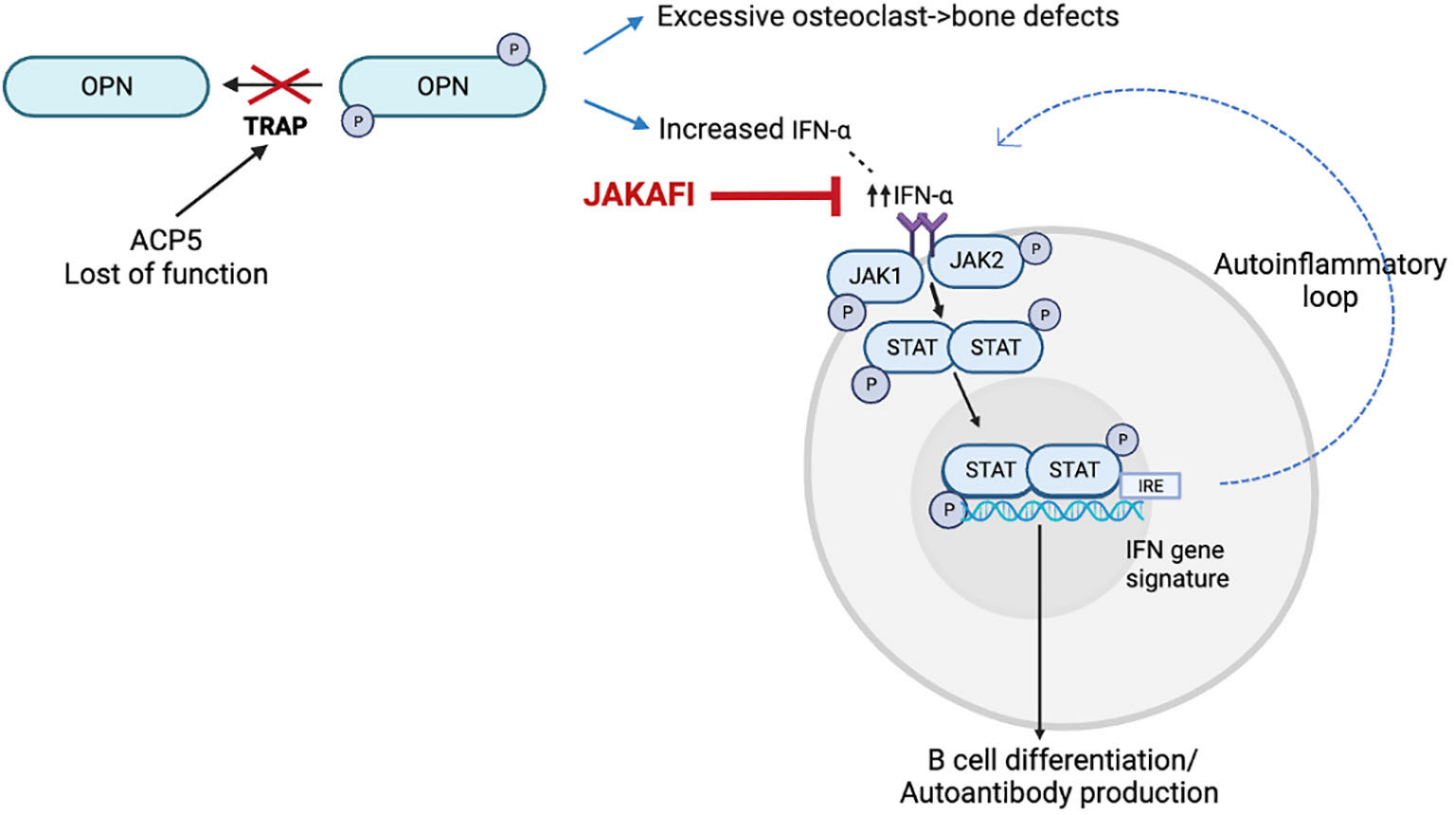
Model for the disease mechanism in SPENCDI, adapted from (6). *ACP5* encodes for tartrate-resistant acid phosphatase (TRAP). Lack of TRAP phosphatase activity results in hyperphosphorylation of OPN. Increased OPN phosphorylation has been linked to increased osteoclast activity, which is believed to contribute to the observed bone and cartilage defects in this disease (6). In antigen presenting cells of the immune system, increased OPN phosphorylation has been associated with increased interferon (IFN)-α release (6). This figure was created using BioRender.com.

SPENCDI (OMIM 607944) is a rare immuno-osseous dysplasia, with an increasing number of affected individuals reported in the literature (1–9). Clinically, the syndrome exhibits a broad phenotypic variability, but most patients present with skeletal and immune manifestations. Adaptative immune dysregulation and autoinflammation comprise the prevailing immune phenotype (1–4, 6–8) although immunodeficiency has also been reported (5, 9). In addition, SPENCDI can manifest with neurologic symptoms (1, 2, 4, 6, 8). Immune dyscrasias, most commonly manifesting as cytopenias and SLE-like symptoms (10–12), are often recalcitrant and refractory to conventional immunomodulatory therapies (1, 2, 6, 10, 13). SPENCDI is an autosomal recessive disease secondary to biallelic pathogenic variants in the *ACP5* gene. *ACP5* encodes for tartrate-resistant acid phosphatase (TRAP). In patients with SPENCDI, *ACP5* mutations lead to abolished TRAP activity in the serum and increased phosphorylation of osteopontin (OPN). OPN has emerged as a possible unifying mediator, as it is expressed in osteoclasts and in cells of the immune system (**Figure 1**). Increased OPN phosphorylation has been linked to increased osteoclast activity, which is believed to contribute to the observed bone and cartilage defects in this disease (6). In antigen presenting cells of the immune system, increased OPN phosphorylation has been associated with increased interferon (IFN)-α release (6). Increased levels of IFN-α may contribute to both, cell autonomous largely autoinflammatory manifestations propagated by innate immune cells as well as non-cell-autonomous systemic autoimmunity in which deregulatory inflammatory signals adversely affect adaptive immune responses.

The constellation of clinical symptoms and biomarkers, i.e. increased type I interferon (IFN) levels and upregulated expression of interferon stimulated genes (ISGs) suggest that SPENCDI is a type I interferonopathy (1, 2, 6, 14, 15). Janus kinase (JAK) inhibitors, also known as jakinibs, are immunomodulators that inhibit the activity of one or more of the JAK enzyme family. The JAK inhibitors ruxolitinib and baricitinib inhibit JAK1 and JAK2 activity downstream of the type I interferon receptor. JAK inhibitors have shown therapeutic benefits in patients with type I interferonopathy, such as SAVI (stimulator of IFN genes–associated vasculopathy with onset in infancy), CANDLE (chronic atypical neutrophilic dermatosis with lipodystrophy and elevated temperature), and Aicardi Goutières syndrome (AGS), as well as patients with undefined interferonopathies (16–18). Two of the patients with higher initial IFN signatures and undefined interferonopathies improved significantly, suggesting that genotype was not necessary to tailor treatment decisions (6). We therefore reasoned that our two patients with SPENCDI may also benefit from targeted JAK inhibition. Here, we report the favorable clinical and biological response of two patients with SPENCDI and recalcitrant cytopenias to ruxolinitb therapy.

## Results

### Patient 1

**Figure 2:**
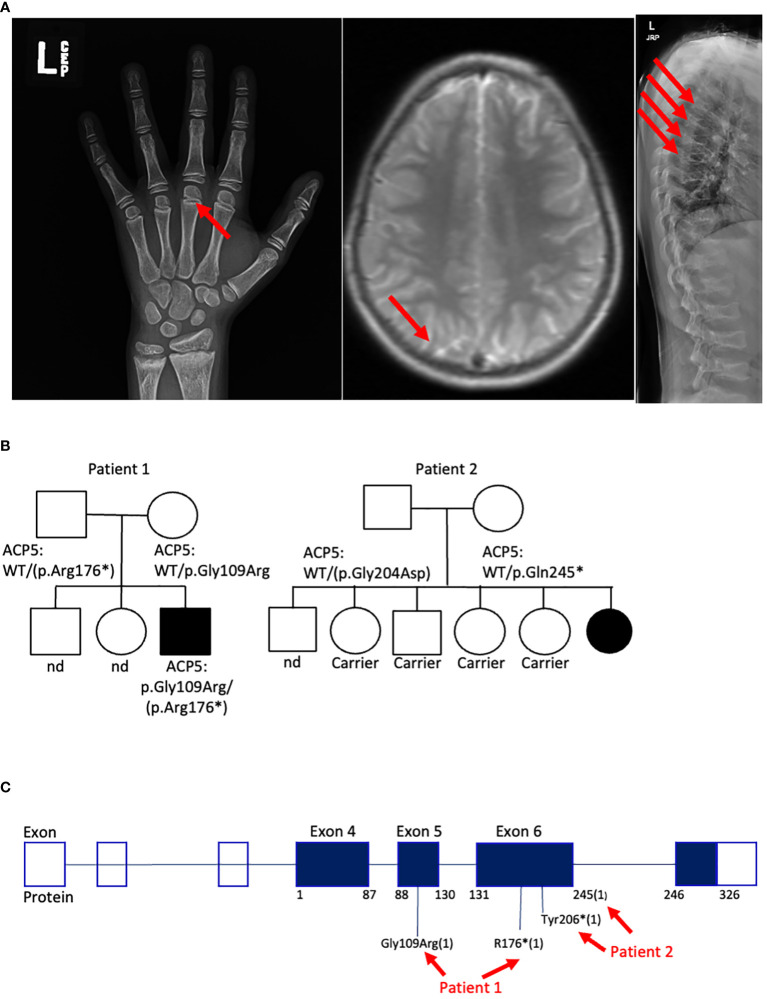
Genetic and clinical characteristics of SPENCDI patients. **(A)** Xray of the left hand (patient #1). Well-defined metaphyseal irregularity (indicated by red arrow) and sclerosis of the distal radius and ulna consistent with bone growth abnormality. Brain MRI (patient #1): nonspecific calcifications of the globus pallidus bilaterally without acute intracranial abnormality (indicated by red arrow). Xray of spine (patient #1): Multilevel compression deformities of the thoraco-lumbar spine (indicated by red arrows). Nd: not determined. **(B)** Family pedigree for patient #1 and patient #2. **(C)** Diagram illustrates the distribution of the reported *ACP5* variants for patient #1 and patient #2. * : stop codon.

is a 19-year-old male who initially presented at 3-years of age with short stature, hypothyroidism (Hashimoto’s disease), and Evans syndrome, manifesting with autoimmune hemolytic anemia (AIHA), thrombocytopenia and neutropenia (**Table 1**). He had been glucocorticoid-dependent since diagnosis. Although his AIHA had initially been responsive to prednisone, the subsequent discovery of multiple vertebral compression fractures (**Figure 2A**) led to discontinuation of glucocorticoids due to concerns for bone metabolism side effects. These therapeutic considerations preceded the diagnosis of SPENCDI with osseous dysplasia as a possible alternative explanation for the vertebral abnormalities. Various other combinations of immunosuppressive therapies including immunoglobulins (IVIG), rituximab, sirolimus, bortezomib and mycophenolate mofetil (MMF) failed to improve his cytopenias or caused severe side effects, such as transaminitis (sirolimus) and anaphylaxis (bortezomib), ultimately necessitating their discontinuation. Further details on presentation and treatment are listed in the **Supplementary Materials**. After a brief period of clinical stability on MMF, the patient’s AIHA eventually recurred, requiring prolonged courses of oral steroids, and prompting referral to immunology for evaluation of an underlying immune dysregulation syndrome.

**Table 1:** Clinical features, laboratory parameters and treatments rendered prior to ruxolitinib therapy for patients #1 and #2.

	Patient #1	Patient #2
**Ethnicity**	Hispanic	Hispanic
Age of onset	3 yo	4 yo
Short stature	Yes	Yes
Developmental delay	No	Yes
Infections	No	Yes (pneumonia)
Hepatosplenomegaly	Yes	Yes
Autoimmunity	Yes	Yes
Thyroid dysfunction	Yes	Yes
Autoantibodies (ANA, DNA, myeloperoxidase, and thyroglobulin antibody)	Positive ANA	Positive thyroglobulin antibody
Cytopenia	Evans syndrome: ITP, AIHA, neutropenia	Evans syndrome: ITP, AIHA, neutropenia
Laboratory parameters at time of presentation		
CD4 T cells (/uL) (NR: 300-2,000)	216	236
CD8 T cells (/uL) (NR: 300-1,800)	223	954
CD19 Cells (/uL) (200-1,600)	247	<20 (s/p Rituximab)
CD56CD16 cells (/uL) (92-1,200)	73	40
IgG (mg/dL) (NR: 440-1,470)	3,460	761 (on IVIG)
IgA (mg/dL) (NR: 31 – 180)	169	<8
IgM (mg/dL) (NR: 25-190)	87.2	272
Titer to Diphtheria (NR: positive)	Positive	Positive
Titer to Tetanus (NR: positive)	Positive	Positive
Titers to Pneumovax	21/23	
ESR (mm/h) (NR: 0-10)	78	
CRP (mg/dL) (NR<0.50)	1.2	1
Bone marrow biopsy	Normal cellularity	Hypercellularity
Xray	Metaphyseal dysplasia and platyspondyly	Metaphyseal dysplasia and platyspondyly
Brain MRI	Calcification of the bilateral globus pallidus	Symmetrical bilateral basal ganglia calcifications, bilateral cerebral subcortical calcifications, and symmetrical bilateral cerebellar calcifications
Cytokine testing (Luminex)		
IFNa2 (pg/mL)	136.8	224.69
IL-1Ralpha (pg/mL)	17008398.6	521.06
IL-6 (pg/mL)	3421.89	9.38
IL-18 (pg/mL)	NA	490.84
TNF-alpha (pg/mL)	1052.83	25.93
Therapy prior to ruxolitinib treatment	Prolonged therapy with steroids, sirolimus, rituximab, IVIG, bortezomib, MMF	Prolonged therapy with steroids, rituximab, IVIG, MMF

**Figure 3:**
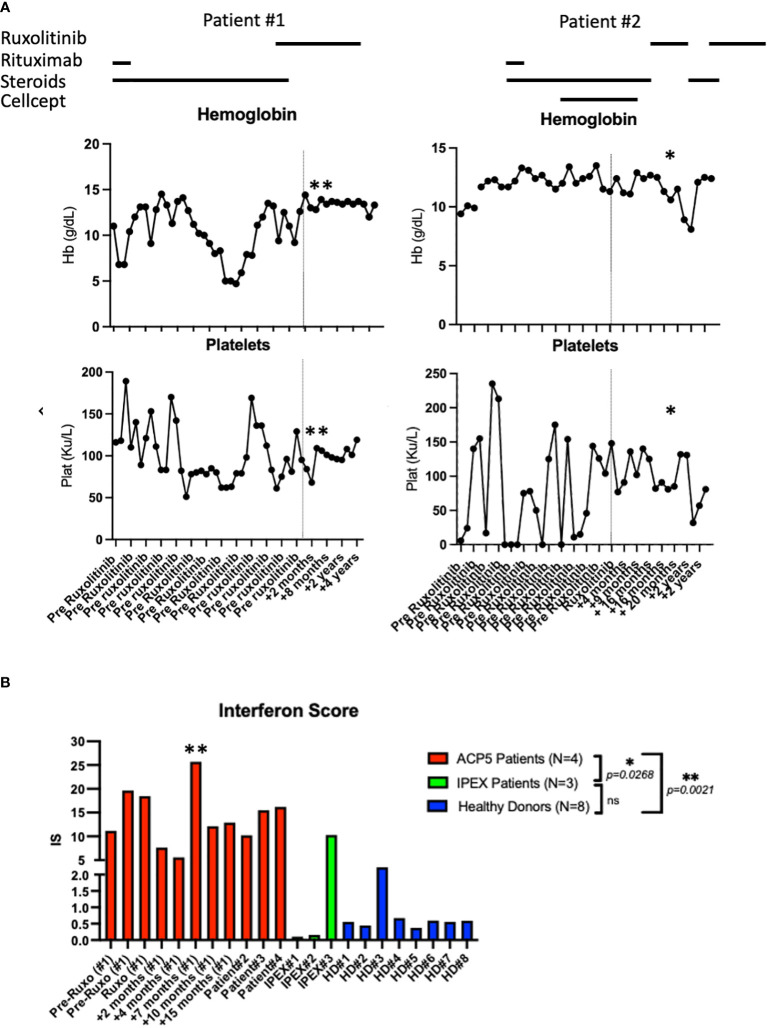
Laboratory parameters and interferon scores pre- and post-ruxolitinib therapy. **(A)** Hemoglobin and platelet count pre and post therapy with ruxolitinib in patient #1 and patient #2. Asterix (*) indicates time of hospital admission for acute viral and bacterial pneumonia complicated by acute SPENCDI flare. Asterix (**) indicates ISG expression in patient #1 transiently spiked during an infectious trigger while he remained clinically symptom free **(B)** Interferon Score of a panel of interferon stimulated genes in 4 SPENCDI patients at various time points throughout treatment, three subjects with IPEX and eight healthy donors. Dashed lines indicate the start of ruxolitinib. HD: healthy donors, Pat: patient, pre: pre ruxolitinib, post: post ruxolitinib, ruxo: ruxolitinib, IS: Interferon Score. Grouped statistical analysis performed using One-Way ANOVA (Kruskal-Wallis test) with Dunn’s multiple comparison test. p-value between groups indicated on the graph. Overall p-value: <0.0001. Level of significance (p)=0.05. Additional information on ACP patient #3 and #4 is detailed in **Supplementary Table 3**.

Trio whole-exome-sequencing (WES) revealed biallelic compound heterozygous pathogenic variants in the *ACP5* gene (c.325G>A, p.Gly109Arg/c.526C>T, p.Arg176*) establishing the diagnosis of spondyloenchondrodysplasia with immune dysregulation (SPENCDI). Both parents were identified as heterozygous asymptomatic carriers of one of the variants (**Figures 2B, C**; **Supplementary Materials**; **Supplementary Table 1**). Based on the favorable therapeutic response of other patients with type I interferonopathies to JAK inhibitors (3, 4, 12, 19–21), and the shared pathophysiologic hallmarks of SPENCDI with other type I interferonopathies (14, 22), a therapeutic trial with the JAK1/JAK2 inhibitor ruxolitinib was initiated (0.4 mg/kg/day). Within a week from initiation of therapy, the patient reported significantly improved energy levels. Within three months, the patient’s anemia and thrombocytopenia were brought into remission and he has remained clinically stable with red blood cell indices in the normal range and platelet counts of approximately 100,000 per microliter of blood without any other additional therapies for the following three-and-a-half years (**Figure 3A**; **Supplementary Table 2**).

While on treatment with ruxolitinib, the patient has not experienced any significant infections. His shorter stature compared to his siblings is likely a sequela of his underlying disease, possibly confounded by early and prolonged glucocorticoid use. He remains neurologically asymptomatic and is performing well academically. Upon transfer to our care, an initial screening MRI brain was obtained which showed nonspecific findings, including calcifications of the globus pallidus bilaterally, but no acute intracranial abnormalities. A subsequent brain MRI three years later showed stable calcifications but no additional abnormal findings. He was immunized with the mRNA COVID-19 vaccine and did not experience any side effects. Despite being fully vaccinated and boosted against COVID-19, he contracted the infection but remained asymptomatic without flaring of his autoimmunity.

In the context of a slowly but steadily rising serum IgG level over 36 months, a bone marrow biopsy screening was obtained which revealed a monotypic population of kappa light-chain restricted plasma cells without an overall increase in blasts. This monotypic population, which lacked cytogenetic abnormalities, encompassed 20% of all bone marrow cells. The patient was subsequently referred to an oncologist and diagnosed with smoldering multiple myeloma. These findings prompted a review of the literature that revealed that approximately half of the patients with SPENCDI also report hypergammaglobulinemia (2, 4, 12, 13, 20, 22).

### Patient 2

is an 8-year-old female who was diagnosed with systemic lupus erythematosus (SLE) at the age of 5 years old. The patient presented with short stature and developmental delay. At diagnosis, the patient suffered from mucocutaneous disease (petechia, purpuric rash), anemia, thrombocytopenia, and splenomegaly. The patient also had, elevated inflammatory markers [ESR: 78 mm/h (normal range (NR):0-15mm/h); CRP:1.2 mg/dL(NR:<0.3 mg/dL)], severely decreased complement levels [CH50: 0 (NR: 42-95 U/mL); C3: 41 mg/dL (NR: 89-173 mg/dL); C4: <2.9mg/dL (NR: 17.0-42.0 mg/dL)], hypergammaglobulinemia, positive ANA titer (>1:1280), elevated dsDNA antibodies (>300 IU/mL), and elevated myeloid peroxidase (MPO) antibodies (**Table 1**). There was no evidence of renal involvement. Cytopenias were moderately responsive to glucocorticoid therapy, but refractory to rituximab and IVIG. At the time of initial assessment in immunology clinic, the patient was treated with glucocorticoids and MMF. Further details on presentation and treatment are listed in the **Supplementary Materials**.

Genetic analysis revealed that the *ACP5* gene had a pathogenic variant with a premature stop codon (c.733C>T, p.Gln245*) and a variant of unknown significance (VUS) resulting in an amino acid substitution (c.611G>A, p.Gly204Asp). Both parents were identified as heterozygous asymptomatic carriers of one of these variants establishing that the patient was compound heterozygote for these two variants (**Figures 2B, C**; **Supplementary Materials**; **Supplementary Table 1**). Given these findings, the patient was assessed for the presence of metaphyseal dysplasia. X-rays of hands and spine showed metaphyseal dysplasia and platyspondyly. Additionally, characteristic radiolucent metaphyseal and vertebral lesions were identified. Apart from an episode of pneumonia at age 4 years, there was no significant history of infections. Due to unclear developmental delay, the patient underwent a screening MRI of the brain which was normal. Considering the positive outcome observed in patient #1, we initiated treatment with ruxolitinib at a dosage of 0.4 mg/kg/day and this patient also showed significantly improved energy levels within one week and improved platelet counts within less than one month from starting treatment. She remained clinically stable and was steroid-free for the next 2-and-a-half years until she developed acute respiratory failure due to parainfluenza and rhinoviral pneumonia with bacterial superinfection and sepsis. Ruxolitinib was discontinued and the patient experienced, after a few days, an acute flare of ITP treated with glucocorticoids. After resolution of sepsis and following the reinitiation of ruxolitinib therapy, the patient’s platelet count improved within 4 weeks (**Figure 3A**).

There is no specific biomarker for tartrate-resistant acid phosphatase activity, however, increased expression in interferon stimulated genes (ISG) has consistently been found in SPENCDI patients. The interferon score of both patients with SPENCDI in our study (patient 1 and 2) was significantly elevated at baseline compared to healthy controls and patients with IPEX (immune dysregulation, polyendocrinopathy, enteropathy, X-linked) syndrome, where autoimmunity is caused by a regulatory T cell defect (**Figure 3B**; **Supplementary Figure 1**). As previously reported by Fremond et al., we only observed a minimal decrease in interferon score in patient #1 following the initiation of ruxolitinib therapy (**Figure 3B**) (23). However, the resolution of cytopenias, combined with the increase in energy, academic performance and general wellbeing associated with ruxolitinib treatment in both patients suggests that interferon scores fail to adequately capture the clinical response. Notably, ISG expression in patient #1 transiently spiked during an infectious trigger while he remained clinically symptom free (**Figures 3A, B**; **Supplementary Figure 1**).

## Discussion

There is growing evidence that SPENCDI is a type I interferonopathy that shares clinical features with other interferonopathies, including autoimmune and autoinflammatory manifestations (1–9). Specifically, a clinical presentation consistent with SLE, in patient #2, should prompt consideration for further genetic testing as monogenic forms of SLE have been described in association with variants in *C1Q, C1R, C1S, CA, DNASE1*, *TREX1* and *ACP5* (11, 12, 24). Genetic testing was pursued for this patient because of the severe and refractory nature of Evans’s syndrome (25–28).

The long-term prognosis and quality of life in patients with SPENCDI is influenced by multiple factors. The severity of the immune dysregulation and the side effects of conventional immunosuppressive therapies play an important role. The use of JAK inhibition in interferonopathies (14) aims at achieving immunomodulation, i.e., a normalization of the augmented immune response by downregulating the signal that is downstream of the type I interferon receptor; which is expected to ameliorate both, autoinflammatory and adaptive autoimmune manifestations alike. Patient #1 has experienced multiple vertebral compression fractures, likely associated with his primary disease but possibly confounded by the long-term glucocorticoid use. The two patients reported in our study were refractory to conventional immunosuppressive therapies but responded to ruxolitinib within only a few weeks from starting treatment, while their interferon score remained elevated. One plausible explanation for this discrepancy could be the consequence of JAK inhibitors on the B cell differentiation by blocking the effects of pro-inflammatory cytokines. JAK inhibitors are known to inhibit the differentiation of human B cells into plasmablasts in response to type I interferon stimuli, thereby reducing the levels of autoantibodies (29, 30). While JAK inhibitor treatment did yield significant improvements in autoimmune cytopenias, it is possible that genes associated with type I interferonopathy remain activated in immune cells with cell-intrinsic defects. Importantly, both patients are tolerating ruxolitinib well, with no significant adverse effects, except for mild neutropenia in patient #1 which constitutes a manageable dose limiting toxicity. Ruxolitinib has been associated with various side-effects including myelosuppression, increased risk of viral infections, transaminitis and non-melanoma skin malignancy. Patients on prolonged ruxolitinib treatment can also develop treatment resistance (31). Both patients were regularly monitored for Epstein-Barr virus, cytomegalovirus, JC, and BK virus reactivation by PCR. which remained negative throughout treatment with ruxolitinib. Ruxolitinib discontinuation syndrome has been documented among myelofibrosis patients, manifesting after the cessation of ruxolitinib treatment. The reported rebound effects vary significantly in severity, ranging from mild to potentially life-threatening symptoms. Abrupt discontinuation of ruxolitinib in myelofibrosis patients may lead to conditions such as worsening of cytopenia, splenomegaly, spleen rupture or acute respiratory syndrome, affecting approximately 15% of individuals (32). The patients and their families have been educated about the risk of rebound if ruxolitinib is abruptly discontinued.

An increase in IgG levels in patients with SPENCDI has been observed by us and others (2) and ultimately lead to the diagnosis of smoldering multiple myeloma in patient #1. We therefore recommend SPENCDI patients should be monitored prospectively for the development of monoclonal gammopathy and regular assessment of immunoglobulin levels, free kappa/lambda light chain ratio and 24 hours urine analysis for Bence-Jones proteinuria should be considered. Any abnormal findings may warrant a referral to the hematologist to assess the need for a bone marrow biopsy.

To the best of our knowledge, there have been no reports of multiple myeloma in patients with SPENCDI. It’s important to highlight that MM and MGUS have also been observed in individuals with VEXAS (Vacuoles, E1 enzyme, X-linked, Autoinflammatory, Somatic) syndrome, a condition propelled by somatic myeloid mutations resulting in autoinflammatory responses (33, 34). In a comprehensive study involving 116 French patients diagnosed with VEXAS syndrome, MDS (myelodysplastic syndrome) was detected in 58 out of 116 cases (50%). Out of these 58 MDS cases, 12 exhibited MGUS (17%) (35). Nevertheless, there remains uncertainty regarding whether somatic UBA1 variants directly instigate MM’s development or whether these variants contribute to conditions like MGUS and plasma cell disorders.

We carefully assessed the potential involvement of the JAK inhibitor therapy in the pathogenesis of multiple myeloma in patient #1. Notably, the patient’s hyperglobulinemia predated the initiation of ruxolitinib treatment. Furthermore, multiple myeloma is not a malignancy associated with either primary T-cell immunodeficiency or prolonged immunosuppressive therapy. There is no documented association between STAT-1 loss-of-function and multiple myeloma (36). In contrast, recent clinical approaches have demonstrated some success in treating refractory multiple myeloma using a combination of ruxolitinib, corticosteroids, and lenalidomide (37). Given the scarcity of literature specifically addressing baricitinib in the context of multiple myeloma, we have elected to continue treatment with ruxolitinib in patient #1.

Another important consideration for the long-term prognosis in patients with SPENDCI is the development of neurologic symptoms in the context of nonspecific brain calcifications. Despite exhibiting bilateral calcification of the globulus pallidus on the brain MRI, patient #1 did not display any neurological symptoms. By contrast, patient #2 exhibited developmental delay but no calcifications noted in the brain MRI. Notably, the neurological status of both patients has remained stable on ruxolitinib treatment for more than 2 years. One might speculate that mitigating systemic inflammation with ruxolitinib could also have beneficial effects on the neurologic manifestations in SPENCDI as has been shown for patients with type I interferonopathy due to Aicardi-Goutières syndrome (38).

The presented cases by us and others (3, 4, 12, 19–21) support the use of JAK-inhibitors as an effective targeted therapy for patients with SPENCDI and severe cytopenias. Lack of effective biomarkers to capture the clinical response to JAK-inhibitors (e.g. IFN scores) highlight the need for further studies to delineate the exact underlying disease mechanism in SPENCDI. SPENCDI patients should be monitored prospectively for the development of monoclonal gammopathy. Further randomized controlled trials will be needed to confirm these findings in larger patient cohorts and evaluate possible neuroprotective benefits of targeted JAK-STAT inhibition in patients with SPENCDI and other interferonopathies.

## Methods

### Laboratory testing

T, B and NK subsets as well as immunoglobulin levels (IgA, IgM and IgG levels) were measured by flow cytometry in the CLIA-certified clinical core laboratory at Stanford (Stanford, CA, USA).

### Cytokines and chemokine testing

IL-18 serum level and CXCL9 were measured by ELISA in a CLIA-certified clinical core laboratory (Cincinnati Children’s hospital, Cincinnati, OH, USA). In **Table 1**, IFNa2, IL-1Ralpha, IL-6, IL-18 and TNF-alpha were measured via Luminex (Human Immune Monitoring Center Stanford, CA, USA). In **Supplementary Table 2**: IL-1β, IL-2 receptor, IL-6, IL-10, IL-18, IFN-gamma, IFN-α were measured by ELISA method (ARUP, Salt Lake City, UT, USA).

### Interferon score

Six out of 15 ISGs were selected based on their expression level in a previous study (15) and measured by quantitative reverse transcription polymerase chain reaction (qRT-PCR) (**Supplementary Figure 1**. In brief, 2.5 mL of blood was collected into PAXgene tubes (PreAnalytix), and total RNA was extracted from whole blood using a PAXgene RNA isolation kit (MagMAX). qRT-PCR analysis was performed using the TaqMan Universal PCR Master Mix (Applied Biosystems) and cDNA derived from 40 ng of total RNA and TaqMan probes for the ISGs *IFI27* (Hs01086370_m1), *IFI44L* (Hs00199115_m1), *IFIT1* (Hs00356631_g1), *ISG15* (Hs00192713_m1), *RSAD2* (Hs01057264_m1), and *SIGLEC1* (Hs00988063_m1). Taqman probes for *HPRT1* (Hs03929096_g1) and *18S* (Hs999999001_s1) ribosomal RNA was used for internal normalization of the PCR assay (Applied Biosystems qpcr analysis module). Delta delta Ct method was used for relative quantification (RQ) of gene expression for each of the six probes, against a single reference control sample. Subsequently, Interferon Score (IS) was calculated by the fold change in the median of the six probes per sample compared to the median of all the healthy controls (n=8).

## Data availability statement

The raw data supporting the conclusions of this article will be made available by the authors, without undue reservation.

## Ethics statement

The studies involving humans were approved by Stanford, Research of compliance, CA, USA. The studies were conducted in accordance with the local legislation and institutional requirements. Written informed consent for participation in this study was provided by the participants’ legal guardians/next of kin. Written informed consent was obtained from the individual(s) (patient 1) and minor(s)’ legal guardian (patient 2) for the publication of any potentially identifiable images or data included in this article.

## Author contributions

YG: Data curation, Supervision, Writing – original draft, Writing – review & editing. MN: Writing – review & editing, Data curation. JV: Writing – review & editing. A-MC: Writing – review & editing. EH: Writing – review & editing. KM: Writing – review & editing. BG: Writing – review & editing. DS: Writing – review & editing. BS: Writing – review & editing. LR: Writing – review & editing. AT: Writing – review & editing. KIW: Writing – original draft. DL: Writing – review & editing. RB: Writing – review & editing. KGW: Methodology, Supervision, Writing – review & editing.

## Supplementary material

Supplementary Figure 1 Interferon signature per gene for 8 healthy controls, 3 IPEX patients, and 4 SPENCDI patients.

Supplementary Table 1 ACP5 variants for patients #1 and #2.

Supplementary Table 2 Clinical phenotype and biological parameters of patient #1 pre and post ruxolitinib therapy.

Supplementary Table 3 Clinical phenotype and biological parameters for ACP5 patient #3 and #4.
