# Supplementary material for: Case report: Refractory Evans syndrome in two patients with spondyloenchondrodysplasia with immune dysregulation treated successfully with JAK1/JAK2 inhibition
Source: Front Immunol. 2024 Jan 29;14:1328005. doi: 10.3389/fimmu.2023.1328005 (PMC10859398; doi:10.3389/fimmu.2023.1328005)
Supplement: Supplementary Figure 1 — Interferon signature per gene for 8 healthy controls, 3 IPEX patients, and 4 SPENCDI patients. [file DataSheet_1.docx]

Refractory Evans syndrome in two patients with spondyloenchondrodysplasia with immune dysregulation (SPENCDI) treated successfully with JAK1/JAK2 inhibition.

Yael Gernez, M.D., Ph.D.^1^; Mansi Narula ^2^; Alma-Martina Cepika, M.D., Ph.D.^2^; Juanita Valdes Camacho, M.D.^3^; Elisabeth G. Hoyte, N.P.^1^; Kirsten Mouradian, NP.^2^; Bertil Glader, M.D.^2^ ; Deepika Singh, M.D.^4^; Bindu Sathi, M.D.^5^; Latha Rao, M.D. ^5^; Ana L. Tolin M.D. ^6^; Kenneth I. Weinberg, M.D.^2^; David B. Lewis, M.D.^1^; Rosa Bacchetta, M.D.^2^ and, Katja G. Weinacht, M.D., Ph.D.^2^

**Supplementary material**

**Presentation and prior treatments:**

**Patient 1.** The patient’s medical history encompasses a complex treatment regimen spanning almost two decades. Prednisone was administered over a decade, varying from 0.25 to 2 mg/kg/day, with intermittent adjustments based on hemoglobin and platelet count responses. At the age of 6 year, while on prednisone for two years, rituximab was prescribed as a steroid sparing agent at a weekly dosage of 375mg/m2 weekly for a total of four doses. This led to a temporary normalization of platelet count during treatment, followed by ITP, necessitating an increase in prednisone dosage. At the age of 12 years old, after 5 years of prednisone treatment, sirolimus was initiated for 10 months at an initial dose of 3mg/m2 (trough drug level 10-15ng/ml), later reduced to 1mg/m2 (trough 3-5ng/ml) due to transaminitis, leading to discontinuation while the patient remained on Prednisone. At the age of 14 years old, after 7 years of prednisone, bortezomib was prescribed at 1.3mg/m2 SQ q72hr, but only three of the four planned doses were administered due to a severe reaction necessitating ICU admission after the third dose. At 15 years of age, eight years into prednisone use, mycophenolate (MMF) was initiated at 1500 mg twice daily. Subsequently prednisone was tapered off three months after MMF commencement, but an exacerbation of autoimmune hemolytic anemia prompted the restart of prednisone, leading to referral to the Hemato-Immunology clinic.

At 17 years old, having been on prednisone for a decade and on MMF for two years, the Hemato-Immunology clinic discontinued MMF and initiated ruxolitinib. After a four-month tapering period, prednisone was successfully discontinued after starting ruxolitinib. This patient has been steroid-free for the past 3 and a half years.

**Patient 2.** The patient presented at 4 years old with a rash on the lower extremities, hepatosplenomegaly, joint pain, fever, and severe thrombocytopenia. The rash was reported to be present for 1-2 years prior to initial presentation but worsened 2 months prior to admission. The patient expressed decreased appetite, fatigue and intermittent pain and swelling in knees, ankles, and feet.

The patient’s past medical history (chronologically) starts with a macrocephaly at 6 months old. While records of prior workup were unavailable, the parents reported that the patient was referred to neurology, with no subsequent follow-up. The patient started walking at 14 months old, started putting 2 words together at 3 years old and at 4 years old, spoke about 30 words.

On examination, several palpable purpuric and ecchymosis lesions on the upper and lower extremities and abdomen and blanchable palmar erythema were present. The abdomen was mildly distended, and the spleen was palpable 2 cm below the costal margin. Macrocephaly was observed and decreased muscle tone was present. There was no evidence of joint effusion or limitation in range of motion to indicate clinical synovitis.

Laboratory evaluation demonstrated mild anemia (hemoglobin 11g/dL), thrombocytopenia (20 10*3/mcL), elevated inflammatory markers with (CRP 1.2 mg/dL) and erythrocyte sedimentation rate 62 mm/h, and severely decreased complements (C3 41.5, C4 < 2.9 and CH50 0). The autoimmune workup demonstrated high titer positive ANA >1:1280 with markedly elevated double-stranded DNA antibodies (>300 IU/ml) and hypergammaglobulinemia (IgG 1828). An abdominal ultrasound demonstrated mild ascites and a chest x-ray demonstrated pleural effusion. An MR angiogram of the neck, chest, abdomen, and pelvis did not demonstrate any evidence of medium or large vessel vasculitis.

The patient meets the 1997 ACR classification criteria for systemic lupus with presence of serositis, hematologic disease (thrombocytopenia), immunologic disorder (elevated double-stranded DNA antibodies) and positive ANA. Given the young age at presentation along with macrocephaly and developmental delay, an underlying genetic immune dysregulation syndrome was suspected, and she was referred to the immunology clinic. A genetic workup revealed pathogenic variants in the *ACP5* gene. A further workup for metaphyseal dysplasia was also undertaken and x-rays of hands and spine demonstrated metaphyseal dysplasia and platyspondyly.

Thrombocytopenia was refractory to conventional immunomodulatory treatment including IVIG, MMF, rituximab and the patient remained corticosteroid dependent. The patient developed corticosteroid related side effects including cushingoid facies, weight gain, glucose intolerance and growth retardation. Eventually she was started on ruxolitinib 0.4mg/kg/day with improvement in thrombocytopenia.

Efficacy measures during the two year and three year follow ups for both patients included hematologic indices, reduction in corticosteroid dose, inflammatory markers, metabolic markers, autoantibodies, serum cytokines, pain scores, quality of life, energy levels, and the IFN signature.

**Genetic variants:**

**Patient 1:** Trio whole-exome-sequencing (WES) (Ambry Genetics, Aliso Viejo, CA, USA) revealed biallelic compound heterozygous pathogenic variants in the *ACP5* gene (c.325G>A, p.Gly109Arg/c.526C>T, p.Arg176*) establishing the diagnosis of spondyloenchondrodysplasia with immune dysregulation (SPENCDI).

The c.526C>T, p.R176* alteration located in exon 6 (coding exon 3) of the ACP5 gene, consists of a C to T substitution at nucleotide position 526. This changes the amino acid from an arginine (R) to a codon stop at amino acid position 176. Premature stop codons, specifically early in the sequence, are typically deleterious in nature. Co-segregation analysis of the c.526C>T (p.R176*) alteration revealed that the proband inherited this alteration from his heterozygous father. The variant is not present in general population.

The c.325G>A, p.Gly109Arg is located in exon 5 (coding exon 2) of the ACP5 gene. This alteration results from a G to a A substitution at the nucleotide position 325, causing the glycine (G) at amino acid position 109 to be replaced by an arginine (R). This alteration has been previously observed in both the homozygous state and in the compound heterozygous state with other ACP5 alterations in multiple individuals with ACP5 (2, 6). Co-segregation analysis of the c.325G>A, p.Gly109Arg alteration revealed that the proband inherited this alteration from his heterozygous mother. This variant is not present in general population.

**Patient 2:** PID invitae panel (San Francisco, CA, USA) revealed biallelic compound heterozygous variants in the the *ACP5* gene (c.733C>T, p.Gln245*/c.611G>A, p.Gly204Asp).

The c.733C>T, p.Gln245* alteration consists of a C to T substitution at nucleotide position 733. This changes the amino acid from an arginine (R) to a codon stop at amino acid position 245. This is expected to disrupt the last 81 amino acids of the ACP5 protein (C terminus of the ACP5 protein). Other variant(s) that disrupt this region (p.Ser267*) have been determinate pathogenic (**Lausch et al,** **Nature Genetics 2011**). Premature stop codons are typically deleterious in nature. Parental testing revealed that this pathogenic variant was inherited from his heterozygous mother. This variant is not present in the general population.

The c.611G>A, p.Gly204Asp replaces glycine with Aspartic acid at codon 204 of the ACP5 protein (p.Gly204Asp). The glycine residue is highly conserved and there is a moderate physicochemical difference between glycine and aspartic acid. The variant is present in database (rs775336126, ExAC 0.009%). Parental testing revealed that this pathogenic variant was inherited from his heterozygous father.

The classification of these variants was ascertained through analysis conducted by two commercial genetic testing laboratories (Ambry Genetics for patient 1, and Invitae for patient 2), and endorsed by our genetics colleagues.

**Supplementary Figure 1.** Interferon signature per gene for 8 healthy controls, 3 IPEX patients, and 4 SPENCDI patients.

**
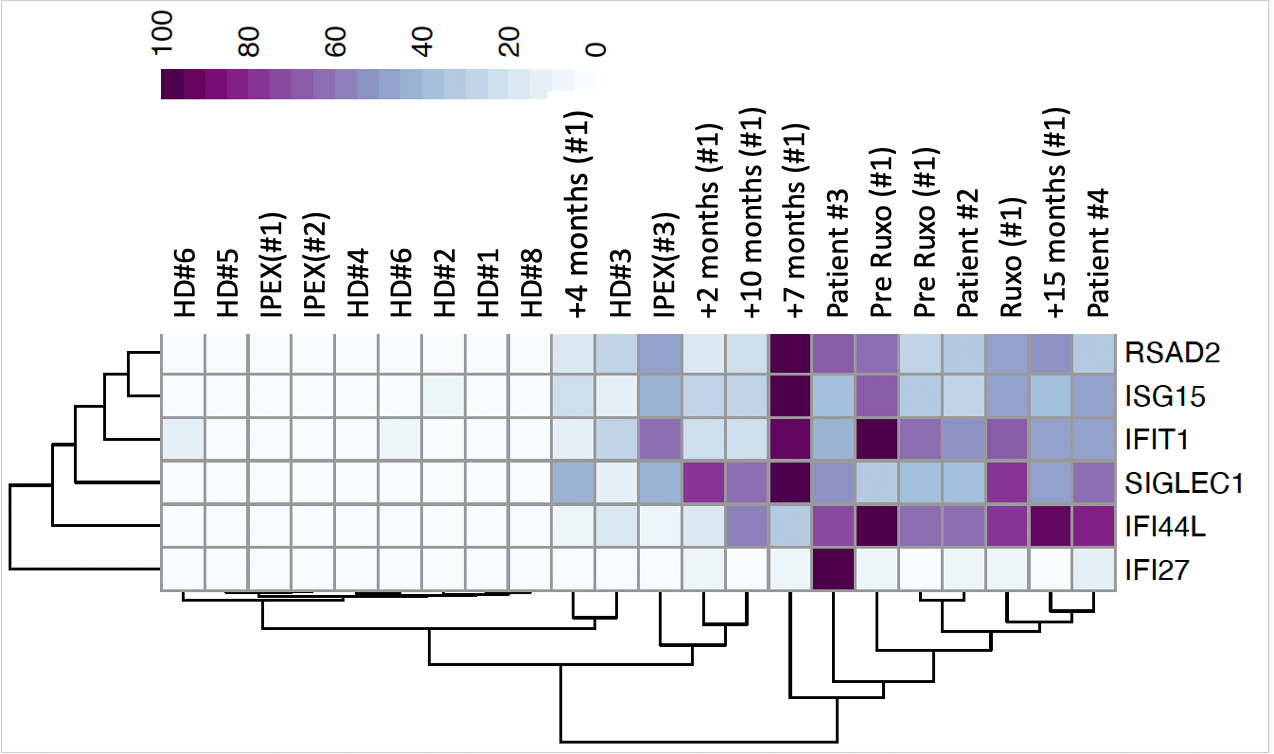
**

Legend: In the heat map, each column (gene) was scaled to its maximum measured value; the scale in the legend is from 0-100%. Both rows and columns are clustered (Manhattan clustering).

Heatmap was made by pheatmap R package. HD: healthy donors.

**Supplementary Table I.** ACP5 variants for patients #1 and #2.

| Patients | Gene | Gene inheritance | Classification | Nucleotide change | Protein change | Alteration type | Alteration classification | Clinic correlation |
| --- | --- | --- | --- | --- | --- | --- | --- | --- |
| Patient #1 | *ACP5* | Heterogenous maternal | Characterized/ Pathogenic | c.325G>A | p.Gly109Arg | Missense | Pathogenic | Positive |
|  | *ACP5* | Heterogenous paternal | Pathogenic | c.526C>T | p.Arg176* | Nonsense | Pathogenic | Positive |
| Patient #2 | *ACP5* | Heterogenous maternal | Characterized/Pathogenic | c.733C>T | p.Gln245* | Nonsense | Pathogenic | Positive |
|  | *ACP5* | Heterogenous paternal | VUS | c.611G>A | p.Gly204Asp | Missense | VUS | Positive |

VUS: variant of unknown significance

**Supplementary Table II.** Clinical phenotype and biological parameters of patient #1 pre and post ruxolitinib therapy.

|  | **Pre ruxolitinib** | **9 mos post- ruxolitinib** | **17 mos post- ruxolitinib** | **21 mos post-ruxolitinib** | **36 mos post- ruxolitinib** |
| --- | --- | --- | --- | --- | --- |
| **Therapy** |  |  |  |  |  |
| Ruxolitinib | - | + (0.4mg/kg/d) | + (0.4mg/kg/d) | + (0.4mg/kg/d) | + (0.4mg/kg/d) |
| Prednisone | + (0.5-1mg/ kg/day) | - | - | - | - |
| MMF | + (2000 mg/ kg/day) | - | - | - | - |
| PJP prophylaxis | + | + | + | + | + |
| **Clinical presentation** |  |  |  |  |  |
| Fatigue | **++** | - | - | - | - |
| **Biological parameters** |  |  |  |  |  |
| WBC (K/uL)  (NR: 4.0-11.0) | 1.8 | 2.2 | 2.2 | 1.6 | 1.7 |
| Neutrophils (K/uL)  NR: 1.70-6.70) | 580 | 890 | 1080 | 730 | 1000 |
| ESR (mm/hr) (NR<15) | 31 | 39 | 44 | 36 | NA |
| Ferritin (ng/mL) (NR<320) | 398.2 | - | - | 268 | 177 |
| IL-1β (pg/mL) (NR : <36) | 86 | 50 | <6.5 | <6.5 | NA |
| IL-2 receptor, soluble (pg/mL) (NR : <1033) | 1490 | 2377 | 693.4 | 780.9 | NA |
| IL-6 **(**pg/mL) (NR:<5) | 7 | 29 | <2 | <2 | NA |
| IL-10 **(**pg/mL) (NR: <5) | 7 | 18 | 18.3 | 22.2 | NA |
| IL-18 (pg/mL) (NR: 89-540) | 1,320 | NA | 1,214 | 1,367 | NA |
| IFN-α **(**pg/mL) | 148.2 | 77.62 | 148 | NA | NA |
| IFN- γ (pg/mL) (NR:<5) | 41 |  | <5 | <5 | <5 |
| CXCL9 **(**pg/mL)  (NR : <121) | 66 | NA | NA | NA | NA |
| **Immune work up** |  |  |  |  |  |
| CD3 T cells /uL (NR: 800- 3,500) | 850 | 843 | 462 | 407 | 402 |
| CD4 T cells /uL (NR: 400-2,100) | 359 | 219 | 191 | 128 | 359 |
| CD8 T cells /uL (NR: 200-1,200) | 478 | 228 | 257 | 234 | 152 |
| B cells/uL  (NR: 200-600) | 332 | 329 | 205 | 128 | 211 |
| NK cells/uL  (NR: 78-470) | 106 NR: 70 - 1,200 /uL) | 17 | NA | 22 | 33 |
| IgG (mg/dL) (NR: 584 - 1,509) | 2,980* | 3,910* | 5,164 | 5,176 | 6,736 |
| IgA (mg/dL)  (NR: 45-234) | 150 | 170 | 181 | 173 | 165 |
| IgM (mg/dL)  (NR: 25-190) | 134 | 136 | 124 | 98 | 88 |

ANA: anti-nuclear antibodies, CRP: C-reactive protein, DAT: direct antiglobulin test; DSDNA: double stranded DNA antibodies, ESR: sedimentation rate, IL for interleukin, LDH: Lactate dehydrogenase, MMF: mycophenolate mofetil, NA: not applicable, NR: normal range, TNF: tumor necrosis factor, IFN: interferon, RBC: red blood cell, WBC: white blood cell. * Normal SPEP, UPEP and free kappa/lambda light chain ratio.

**Supplementary Table III.** Clinical phenotype and biological parameters for ACP5 patient #3 and #4.

|  | Patient 3 | Patient 4 |
| --- | --- | --- |
| Clinical presentation |  |  |
| Age of onset | 6 months | 3 yo |
| Short stature | Yes | Yes |
| Developmental delay | Yes | Yes |
| Infection | No | Yes (pneumonia) |
| Hepatosplenomegaly | yes | Yes |
| Autoimmunity | Yes | Yes |
| Thyroid dysfunction | Yes | Yes |
| Autoimmunity  (ANA, DNA natif) | Negative ANA | Transient and low ANA titers |
| Cytopenia | ITP, AHAI, neutropenia | ITP, AHAI, neutropenia |
| Laboratories |  |  |
| CBC  -Hb (K/mm^3^)  -Plat (K/mm^3^)  -WBC (K/mm^3^)  -ANC (K/mm^3^) | 10.7 | 11 |
|  | 98,000 | 85000 |
|  | 5.6 | 2.3 |
|  |  | 1.74 |
| CD4 T cells (cells/mcL) | 236 | 394 |
| CD8 T cells (cells/mcL) | 954 | 335 |
| CD19 cells (cells/mcL) | <20 (s/p Rituxan) | 32 |
| CD16CD56 cells (cells/mcL) | 40 | 177 |
| IgG (mg/dL) | 761 | 766 |
| IgA (mg/dL) | <8 | 0 |
| IgM (mg/dL) | 272 | 60 |
| Titer to Diphtheria | Positive | Positive |
| Titer to Tetanus | Positive | Positive |
| Titers to Pneumovax | - | Positive |
| ESR (mm/hrs) | - | 16 |
| CRP (mg/dL) | 1 | 0,14 |
|  |  |  |
| Bone marrow biopsy | Hypercellularity | Normal cellularity |
| Xray |  |  |
| Brain MRI | Symmetrical bilateral basal ganglia calcifications some bilateral cerebral subcortical calcifications and symmetrical bilateral cerebellar calcifications | Basal ganglia microcalcifications  (2020) |
| Genetic study | Biallelic variants in ACP5  c.643G>A  (p.Gly215Ar) | Biallelic variants in ACP5  c.791T>A  p.Met264Lys |
|  |  |  |
| IFNa2 (pg/mL) | 224.69 | - |
| ILR1alpha (pg/mL) | 521.06 | - |
| IL-6 (pg/mL) | 9.38 | - |
| IL-18 (pg/mL) | 490.84 | - |
| TNF-alpha (pg/mL) | 25.93 | - |
| Treatment | Chronic steroids, rituximab, cellcept | Chronic steroids, rituximab (2018) sirolimus, IVIG and ruxolitinib |
